# Supplementary figures and images for: Integrated Genomic Analysis Reveals the Impact of Avermectin on Chromatin Structure and Gene Expression Regulation in Bombyx mori
Source: Insects. 2025 Mar 12;16(3):298. doi: 10.3390/insects16030298 (PMC11943398; doi:10.3390/insects16030298)

A

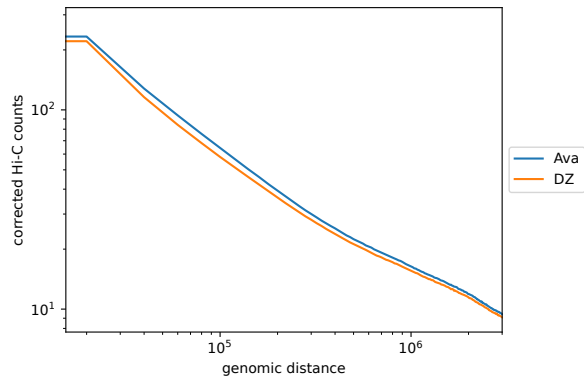

B

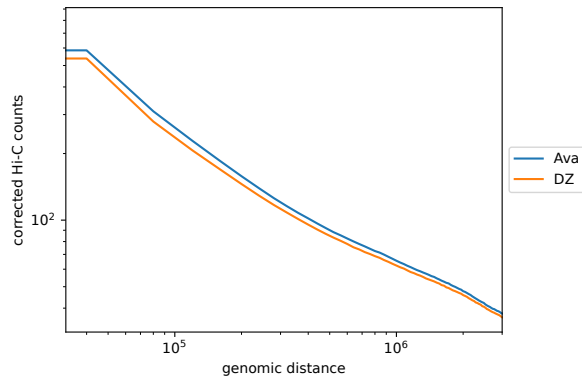

C

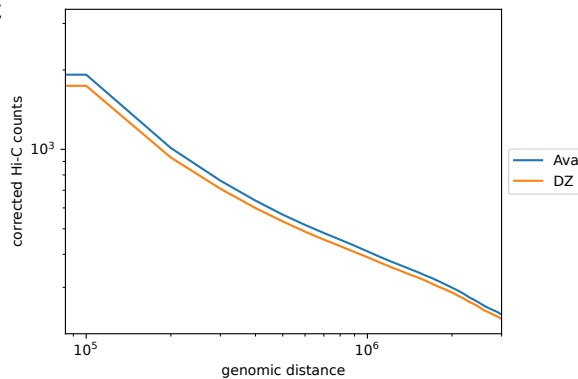

Supplement: Supplementary file 1 [file insects-16-00298-s001.zip › Figure S1.pdf]

A

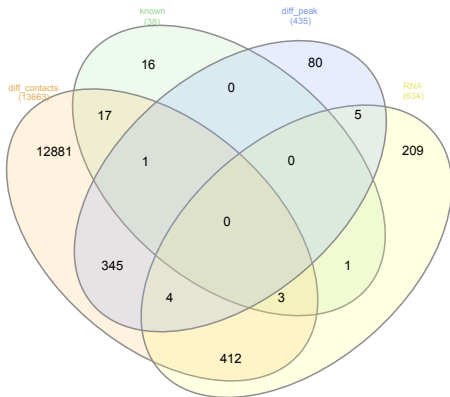

B

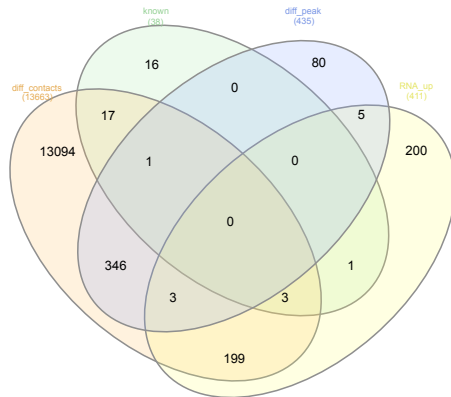

Supplement: Supplementary file 1 [file insects-16-00298-s001.zip › Figure S2.pdf]
